# From thresholds to trajectories: a perspective on reframing alloimmune risk for computational modeling in solid organ transplantation

**DOI:** 10.3389/fimmu.2026.1877220

**Published:** 2026-06-08

**Authors:** Rajdeep Das, Reut Hod-Dvorai

**Affiliations:** 1 Department of Pathology, University Hospitals Cleveland Medical Center, Cleveland, OH, United States; 2 Case Western Reserve University School of Medicine, Cleveland, OH, United States; 3 Department of Pathology, SUNY Upstate Medical University, Syracuse, NY, United States

**Keywords:** alloimmune risk, computational modeling, HLA, machine learning, solid organ transplant

## Abstract

Immunological risk prediction in solid organ transplantation has long depended on threshold-based representation of histocompatibility data: donor-specific antibodies (DSA) reported as positive or negative, mean fluorescence intensity (MFI) assessed by fixed cutoffs, molecular mismatch assigned at transplantation, and assays interpreted at a single time point. Although practical, these conventions simplify the complex and dynamic nature of the immune response. In this perspective, we argue that as machine learning (ML) algorithms and computational approaches enter the field of transplant immunology, we need to focus on whether the inputs that feed into these tools and models reflect how alloimmunity actually behaves. We propose treating alloimmune risk as a time-indexed alloimmune state, updated whenever new data are available, across four domains: antibody profile, molecular mismatch and predicted immunogenicity, recipient immune context, and graft context. Within this framework, the features that experienced clinicians and histocompatibility experts already track (e.g., DSA velocity, persistence, epitope spreading, concordance with graft injury) become computable rather than implicit. We discuss how moving from thresholds to trajectories impacts model design, why HLA laboratory expertise becomes more important rather than less, and why interpretability, regulation, and external validation should precede clinical adoption.

## 1 Introduction: current data structures may not fully capture alloimmune complexity

The alloimmune response after transplantation is shaped by multiple factors, including donor–recipient Human leukocyte antigen (HLA) disparity, prior sensitization, immunosuppression, infection, inflammation, adherence, and graft injury (1, 2). Transplant candidates undergo extensive immunological evaluations, performed by clinicians and HLA laboratories during the pre- and post-transplant phases in order to minimize the risk for rejection and maximize the longevity of the transplanted organ.

**Figure 1:**
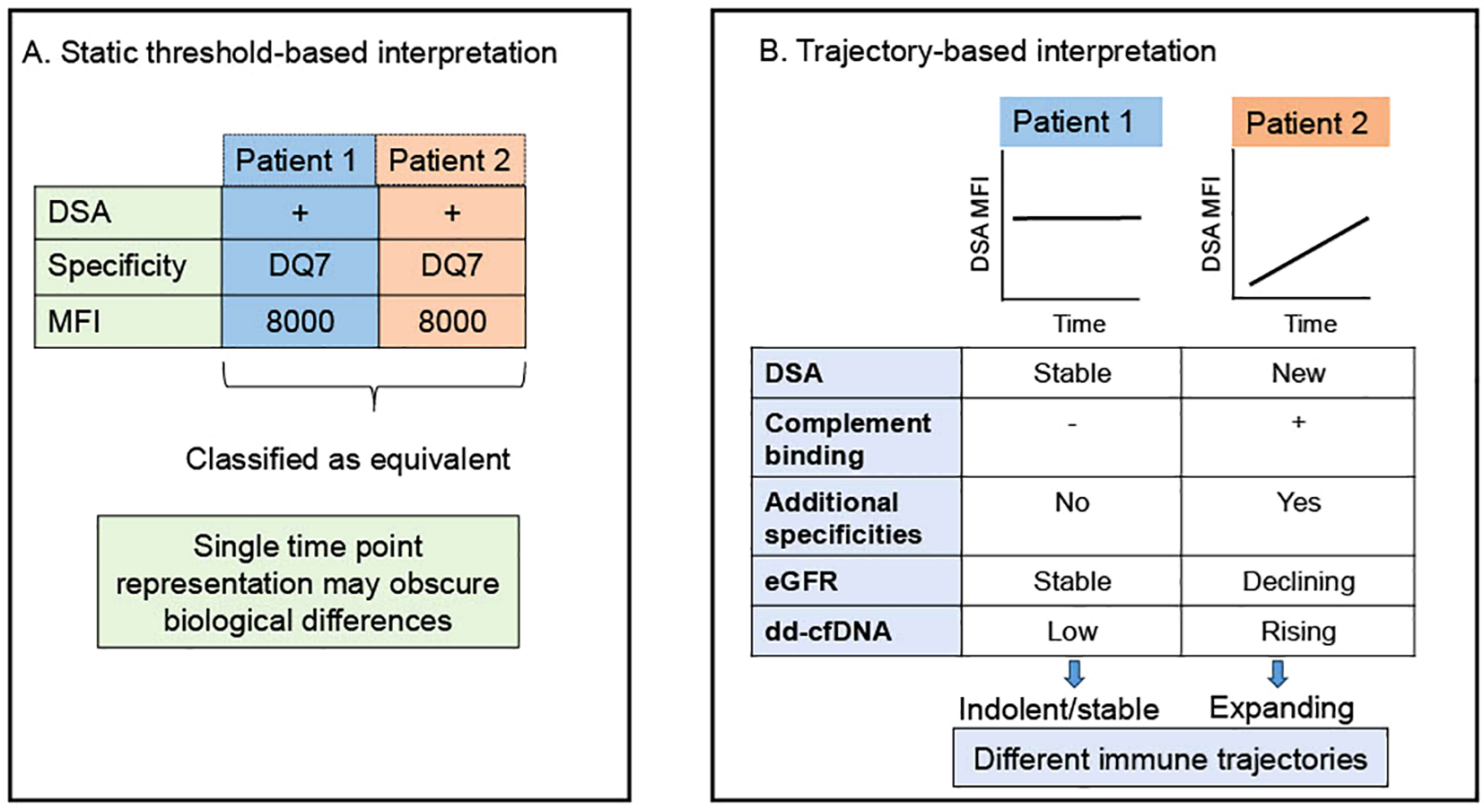
Threshold-based versus trajectory-based interpretation of alloimmune risk. Two kidney transplant recipients, both eighteen months post-transplant, present with anti-HLA-DQ7 donor-specific antibodies at similar mean fluorescence intensity (MFI) values and comparable molecular mismatch scores. **(A)** When evaluated at a single time point, both patients appear immunologically equivalent: DSA (donor specific antibodies) positive, MFI above the institutional threshold, similar molecular mismatch category. **(B)** Longitudinal data tell a different story. Patient 1 has maintained a stable DSA MFI over time, with no complement binding, no new antibody specificities, stable eGFR (estimated glomerular filtration rate), and low dd-cfDNA (donor-derived cell-free DNA) features consistent with an indolent alloimmune state. Patient 2, by contrast, has a newly detected and rising DSA MFI, newly positive complement binding, emerging additional specificities suggesting epitope spreading, declining eGFR, and rising dd-cfDNA. These two patients are not immunologically equivalent; they represent meaningfully different clinical situations that threshold-based data representation fails to distinguish.

To illustrate the complexity of the alloimmune response and the uniqueness of each case, consider the following two kidney transplant recipients: both eighteen months post-transplant, have similar molecular mismatch scores against their donor, and both presenting during routine monitoring with an anti–HLA-DQ donor-specific antibody (DSA) with similar mean fluorescence intensity (MFI) which is above the program’s threshold for positivity. Based on these data alone, they may seem immunologically equivalent. Their DSA is positive, their MFI exceeds an institutional threshold, their molecular mismatch category is already assigned, and their calculated risk, based on a specific time point, places them in a similar clinical tier. However, when considering additional information, these patients represent very different scenarios; the first recipient has been DSA positive for nearly a year with stable MFI and negative complement binding across multiple serial measurements. No new antibody specificities have emerged over time, their estimated glomerular filtration rate (eGFR) is unchanged, and donor-derived cell-free DNA (dd-cfDNA) remains low. The second recipient recently developed the DSA, with MFI values trending up, complement binding is newly positive, and additional antibody specificities, consistent with epitope spreading, have emerged. In addition, the patient’s dd-cfDNA is rising (**Figure 1**). Experienced clinicians and HLA laboratory directors would recognize that these patients represent distinct clinical situations. The first reflects a relatively stable and potentially indolent alloimmune response. The second suggests a trajectory associated with substantially higher risk of antibody-mediated injury. Yet the representations routinely used to report these two scenarios, which relies on binary DSA status (negative/positive), MFI thresholds, single time point tests, and fixed molecular mismatch tiers often fail to distinguish between them.

This, in short, is the problem that this perspective paper is aiming to address. Over the past two decades, the histocompatibility field has made substantial advances in antibody characterization and measurement. Solid-phase single-antigen bead (SAB) assays detect donor-specific HLA antibodies with extraordinary sensitivity (3). Molecular mismatch approaches, including amino acid sequence analysis, eplet/epitope analysis, indirect allorecognition, electrostatic mismatch score, and others, assess donor–recipient disparity at the molecular level rather than serologic type (4–7). However, the true value of antibody measurements depends on longitudinal follow up and interpretation within the context of the immune state. Functional assays, such as those assessing complement binding and/or serial dilutions interrogate properties that may better reflect the pathogenic potential than MFI alone (8–10). Notably, this perspective builds upon and extends prior conceptual frameworks such as the STAR consensus workgroup that framed alloimmunity as a dynamic, multidimensional, and context-dependent process (11, 12).

Yet, for predictive modeling purposes, alloimmune risk is often captured in ways that simplify the complex biology of the alloimmune response. Approaches that are based on collection of static (e.g., single time point) and/or binary (present/absent) data capture important risk elements but are less suited to model how immunologic features change and interact with other clinical factors over time. The challenge is not necessarily lack of data or inadequate algorithms, but that the variables entered into these algorithms may not fully represent longitudinal and context-dependent response. Therefore, we propose a shift from thresholds to trajectories in the setting of algorithm development. Although aspects of this framework may generalize across solid organ transplantation, we focus here on kidney transplantation, where longitudinal alloimmune monitoring is very common and where the limitations of threshold-based thinking are especially visible.

## 2 The persistence and limitations of threshold-based thinking

Thresholds provide clarity, they transform continuous, sometimes noisy, context-dependent measurements into categorical silos that can be communicated, stored, and acted upon. Their practical utility is clear, but utility does not always coincide with biological truth. MFI is a good example, because it is a semi-quantitative signal impacted by bead antigen density, bead composition, assay conditions, inhibitory factors, lot-to-lot variation, and other features of the solid-phase assay chemistry (3). Importantly, it is not a direct measure of antibody concentration, affinity, or pathogenicity (13, 14). Despite this, some clinical protocols and studies assessing risk continue to treat MFI cutoffs as a linear representation of biological quantity (15, 16).

The binary assignment of DSA status reflects another problem. Collapsing an antibody profile into present versus absent disregards information about antibody repertoire, functional characteristics such as complement-binding capability, and broader clinical context, all of which may carry prognostic relevance beyond antibody presence alone (8, 9). Some recipients maintain high-level DSA for years without detectable graft injury, whereas others develop rejection despite antibody levels that would traditionally be considered modest. These are not paradoxes; these are examples of multidimensional biology interpreted through narrower lenses. Alloimmunity is a continuous biological process evolving over time and shaped by the immunological context. Imposing thresholds on such a system is not just a simplification, it may also result in loss of information for downstream applications.

## 3 Alloimmunity as a dynamic biological system

The immune response to an allograft is a dynamic process that includes B-cell activation, affinity maturation, T follicular helper engagement, epitope spreading, etc., and it is shaped by the continuous modulation of these processes by immunosuppression, infections, vaccinations, inflammation, and medication adherence (4, 12). A DSA measurement at a single time point is therefore only a snapshot of a broader biological process. Sequential monitoring is required to assess direction, persistence, acceleration, and pathogenesis.

While molecular mismatch contributes to alloimmune recognition, it does not fully explain how alloimmune injury develops over time. Its clinical relevance depends on the recipient’s evolving immune context (17, 18). For computational modeling, this means that molecular mismatch should be interpreted not as an isolated predictor, but in relation to longitudinal antibody behavior and graft injury markers.

## 4 The alloimmune state: a multidimensional framework

Since alloimmunity is a dynamic process, predictive modeling frameworks should treat it accordingly. We propose considering the patient’s alloimmune state as a time-indexed, multidimensional representation of the immunological interaction between the recipient and the donor, updated whenever new laboratory or clinical data become available to create an immune trajectory that captures changes over time. This approach is how most experienced HLA laboratory directors and transplant clinicians with expertise reason when interpreting test results.

From a computational viewpoint, the alloimmune state can be represented across four domains:

1. Antibody profile: DSA specificity, antibody strength, persistence, presence of historical antibodies, complement binding (when available), dilution behavior, and repertoire breadth (8, 15).
2. Molecular mismatch and predicted immunogenicity: donor-recipient molecular mismatch, eplet load, predicted indirect presentation, and physicochemical or electrostatic disparity (4–6).
3. Recipient immune context: immunosuppression, adherence, infection, vaccination, inflammation, sensitization history, and immune activation.
4. Graft context: time post-transplant, eGFR, Creatinine, proteinuria, dd-cfDNA, biopsy findings when available, gene-expression data, and rejection history (19).

This framework converts clinical interpretation into computable longitudinal features, and when alloimmunity is captured by its dynamic nature it may be easier to predict.

## 5 Immune trajectory: capturing change over time

Experienced transplant clinicians consider trajectories. They recognize when a DSA is rising or decreasing rather than merely present, when new antibodies appear, or when a previously stable antibody changes in response to an infection or medication modulation. In essence, they perform a time-series analysis on biological data. However, this reasoning is only partly reflected in risk studies, which often summarize alloimmune history as baseline, peak, or most recent DSA, rather than modeling the pattern of change itself (8, 15).

**Figure 2:**
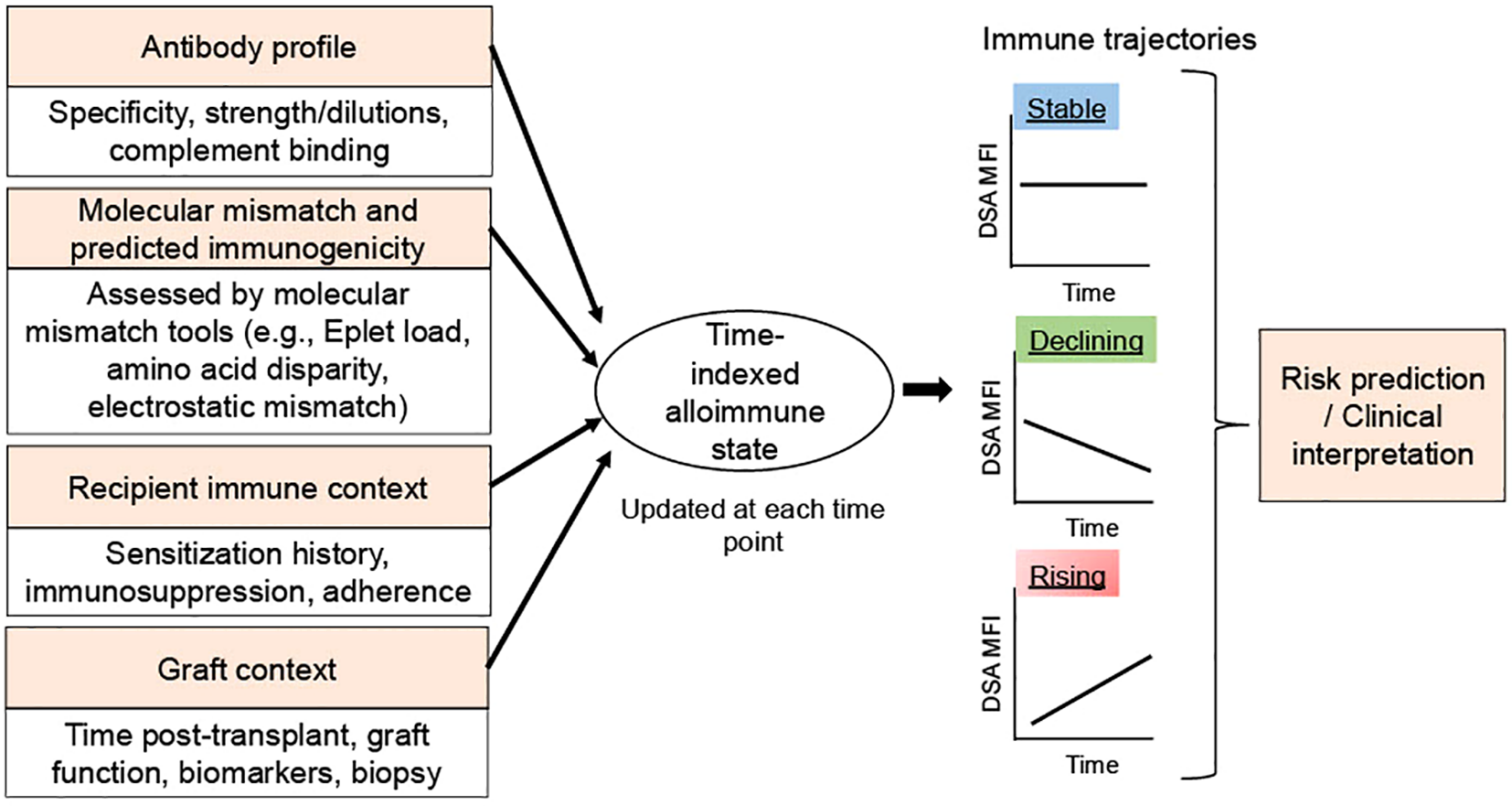
Proposed conceptual framework for representing alloimmune risk as a time-indexed alloimmune state. Rather than capturing alloimmune risk at a single time point, this framework organizes relevant clinical and laboratory data into four domains: antibody profile, molecular mismatch and predicted immunogenicity, recipient immune context, and graft context that together define a time-indexed alloimmune state, updated whenever new data become available. As this state evolves across sequential time points, it generates immune trajectories reflecting the direction and pace of the alloimmune response: stable, resolving, or escalating. These trajectories, rather than isolated measurements, form the basis for risk prediction and clinical interpretation. The framework is intended to make explicit the kind of longitudinal reasoning that transplant clinicians and HLA laboratory directors already apply in practice, and to render it computable for machine learning applications.

A trajectory-based framework asks not what the MFI is, but how quickly it is changing and in what direction; not whether an antibody is present, but whether the antibody repertoire is expanding in a direction predicted by shared epitopes; and not whether a DSA is present, but whether its functional properties are changing in a way consistent with pathogenesis. These distinct approaches are summarized in **Figure 2** and represent measurable questions that can be answered using data that HLA laboratories already generate during routine follow-up.

The trajectory-based approach is already acceptable across different medical disciplines: serial plasma HIV RNA guides assessment of treatment response and virologic failure, while oncology models often use biomarker kinetics rather than isolated valued (20–22). Kidney transplant clinicians and HLA experts similarly interpret DSA trajectories; the opportunity is to structure this reasoning more explicitly for alloimmune risk prediction.

## 6 Computational implications: modeling the process, not a snapshot

Machine learning (ML) algorithms and computational approaches are well suited for analyzing multidimensional immunologic variables, patterns and longitudinal time-series data, and for providing risk prediction. Accordingly, ML-based tools have been applied across all phases of transplantation, including donor assessment, biomarker discovery, rejection classification, immunosuppression monitoring, delayed graft function and outcome prediction. Gene expression–based tools such as Banff Human Organ Transplant (B-HOT) and the Molecular Microscope Diagnostic System (MMDx), as well as ML-based digital pathology approaches, illustrate how computational methods can support renal allograft biopsy interpretation and molecular phenotyping. (23–25). A broader review of ML applications in transplantation is beyond the scope of this article and has been addressed elsewhere (26–29). Here, we focus on how computational tools can improve donor–recipient compatibility assessment by representing alloimmune risk as a dynamic process rather than a static snapshot.

In the realm of transplant immunology, computational and ML algorithms are used to better predict HLA-peptide binding affinity (30, 31), and to predict neo-antigens and model immunogenicity in ways relevant for precision medicine and immunotherapy (32). These algorithms can also assist with identifying patterns in HLA antibody testing which are not easily identified by humans (33), thus enhancing interpretation of complex antibody profiles to support compatibility assessments. Modeling of immune allorecognition can improve transplant risk prediction and donor-recipient matching in the context of both solid organ and stem cell transplants (34, 35). ML-based tools can predict the formation of *de-novo* DSA, and assist with epitope-based matching. For example, one algorithm can identify non-self HLA-derived peptides presented in the context of self-HLA molecules to T-cells, providing a score of T-cell response. Another algorithm can score the number of exposed donor-recipient mismatched amino acids to predict B-cell response. Combining multiple algorithms has been shown to enhance risk stratification in kidney transplantation (36–38).

Importantly, ML models are only as informative as the data used to train them. A sophisticated algorithm trained on static, threshold-based inputs may reproduce the limitations of the methodologies that generated those inputs. If DSA is reported only as present or absent, if MFI is reduced to a single peak value, or if molecular mismatch is treated as a fixed scalar assigned at transplantation, then temporal and contextual information has already been discarded before modeling begins.

In the context of this paper, we envision ML and/or computational tools for histocompatibility assessments that can interface with laboratory information systems and electronic medical record systems to extract the following data: 1) donor and recipient HLA genotypes, preferably at high resolution, with imputation when lower-resolution typing is available; 2) recipient antibody profiles across multiple time points, representing current and historical DSA, specificity, strength, complement-binding (when applicable) and repertoire expansion; and 3) clinical and allograft-related variables such as demographics, sensitization history, underlying disease, immunosuppression exposure, time post-transplant, graft function, rejection history, biopsy findings when available, and donor-derived cell-free DNA. Together, these data could represent each patient as a sequence of time-indexed alloimmune states rather than as a single risk score.

Using this type of framework, clinical observations become computable trajectory-representing features that are able to capture trends (e.g., DSA emergence, persistence, disappearance, velocity of MFI increase, etc.). Methodologically, this kind of trajectory representation lends itself to approaches such as joint models for longitudinal and survival data, survival models with time-varying covariates, and recurrent or transformer-based models for irregularly sampled longitudinal data. The promise of ML in histocompatibility is therefore not only in assisting humans with pattern recognition, but also in formalizing dynamic patterns that expert interpreters already recognize.

## 7 Interpretability and the role of the HLA laboratory

A trajectory-based computational framework does not diminish the role of the HLA laboratory; it relies on close integration with it. The value of these models depends on high-quality laboratory data and expert interpretation. HLA laboratories remain central to recognizing artifacts, assessing donor specificity, and placing antibody patterns into clinical context.

ML-based tools may support HLA antibody interpretation by helping identify recurring reactivity patterns (33, 39), and improving consistency across cases and centers. These applications may be especially useful in highly sensitized patients, where multiple overlapping antibody specificities and epitope patterns can make interpretation challenging (40). These tools can also provide solutions to lot-to-lot variability and outlier detection, including false-positive and nonspecific patterns, resulting in more reliable and consistent antibody assignments (39, 41). However, such tools require large, carefully curated datasets and meaningful benchmarking (42), particularly given inter-laboratory variation in SAB interpretation (14, 43) and the absence of universal thresholds for antibody positivity (14, 40).

Importantly, as ML-assisted compatibility and risk-prediction tools become more accessible, we caution against the premature clinical adoption of “black-box” models that are difficult to interpret. Transparent reporting, thorough external validation, and close integration with HLA laboratory expertise will be essential before these tools can be routinely used for clinical decision-making. Their role should be to support expert judgment, improve consistency, and make complex longitudinal interpretation more scalable, while preserving the central role of the HLA laboratory in assay oversight and clinical context.

## 8 Testable implications and future directions

The proposed framework generates specific empirical questions for future studies. Several testable hypotheses follow from this framework. First, do longitudinal DSA features outperform peak, baseline, or most recent MFI for predicting rejection or graft injury? Second, does molecular mismatch improve prediction abilities when modeled with immunosuppression exposure, inflammatory events, sensitization history, and antibody trajectories? Third, do trajectory-based models retain performance across centers with different SAB interpretation practices and thresholds?

Multicenter datasets linking serial antibody profiles, laboratory interpretation, treatment, graft injury markers, biopsy findings, and clinical outcomes are needed to evaluate these models across different practice settings. Importantly, current and future models should be assessed not only for discrimination, but also for interpretability, clinical utility, fairness, and generalizability. Last, but not least, while incorporating ML models and computational tools present an exciting opportunity to enhance current matching algorithms and organ allocation, we must ensure that future systems and algorithms are equitable, explainable, accountable, and are handled responsibly with appropriate regulations and protection of data privacy (44). Collaboration between HLA professionals, physicians, data scientists and regulatory agencies will be crucial for successful implementation of these tools in our practice.

## 9 Conclusion

As transplant immunology is entering an era in which ML-based prediction tools are becoming more commonly used, we need to shift our thinking from static capture of histocompatibility data into a more dynamic approach. We propose that alloimmune risk should be evaluated in the context of sequential multidimensional states evolving over time. This shift from thresholds to trajectories better reflects how transplant clinicians and HLA laboratory experts already reason when assessing risk. The promise of computational modeling in transplantation will depend not only on larger datasets or more advanced algorithms, but on whether we can represent alloimmune biology in a form that preserves its complex and changing nature. Better data representation can serve as a bridge between measurement and prediction, and between laboratory interpretation and clinical decision-making.

## Data Availability

The original contributions presented in the study are included in the article/supplementary material. Further inquiries can be directed to the corresponding author/s.
